# Supplementary material for: A pandemic-enabled comparison of discovery platforms demonstrates a naïve antibody library can match the best immune-sourced antibodies
Source: Nat Commun. 2022 Jan 24;13:462. doi: 10.1038/s41467-021-27799-z (PMC8786865; doi:10.1038/s41467-021-27799-z)
Supplement: Supplementary file 1 — Supplementary Information [file 41467_2021_27799_MOESM1_ESM.pdf]

A pandemic-enabled comparison of discovery platforms demonstrates a naïve antibody library can match the best immune-sourced antibodies

Fortunato Ferrara [1], M. Frank Erasmus[1], Sara D'Angelo[1], Camila Leal-Lopes[2], André A. Teixeira[2], Alok Choudhary[3], William Honnen[3], David Calianese[3], Deli Huang[4], Dennis R. Burton[5,6], Abraham Pinter[3], Andrew R.M. Bradbury[1]*.

[1]Specifica Inc, Santa Fe, NM, 87505, USA.
[2]Bioscience Division, New Mexico Consortium, Los Alamos, NM, 87544, USA.
[3]Public Health Research Institute, New Jersey Medical School, Rutgers, The State University of New Jersey, Newark, NJ 07103, USA.
[4]Life Sciences Institute, Zhejiang University, Hangzhou, China.
[5]Department of Immunology and Microbiology, The Scripps Research Institute, La Jolla, CA 92037, USA.
[6]Ragon Institute of MGH, MIT and Harvard, Cambridge, MA 02139, USA

*Corresponding author. Email: A.R.M.B. (abradbury@specifica.bio)

– Supplementary Information –

Supplementary Figure 1. Gating strategy for yeast sorting.

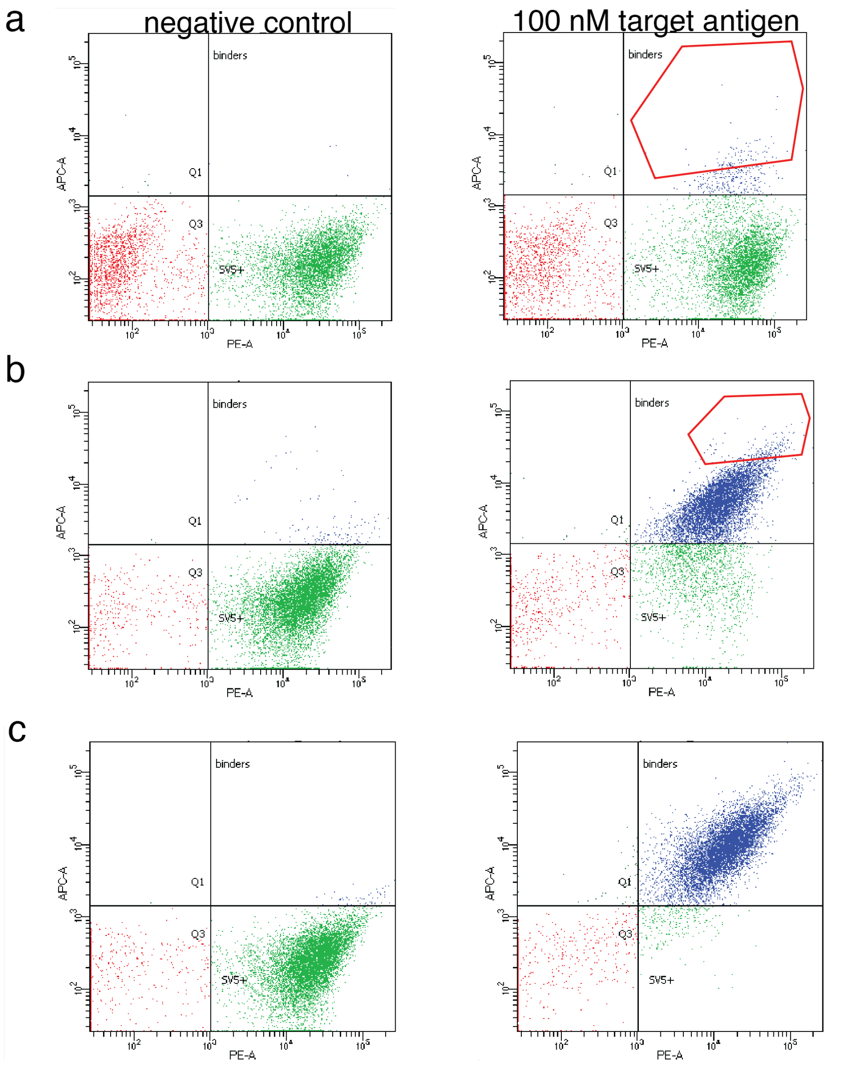

The yeast cells were analyzed simultaneously for: i) display levels (x axis) determined by the binding of anti-V5 PE-conjugated antibody to the V5 tag found downstream of the scFv; and ii) target binding by the scFvs (y axis) detected by APC conjugated streptavidin binding to the biotinylated antigen. The "binders" gate represents double positive cells which display scFvs and bind to the target and correspond to ~0.5-1% of the scFv-displaying yeast cells. The resulting population after two rounds of sorting show that all the displaying yeast cells bind to the specific antigen and not the negative control one.

Supplementary Figure 2. Schematic representation of the batch reformatting strategy from scFvs to IgGs.

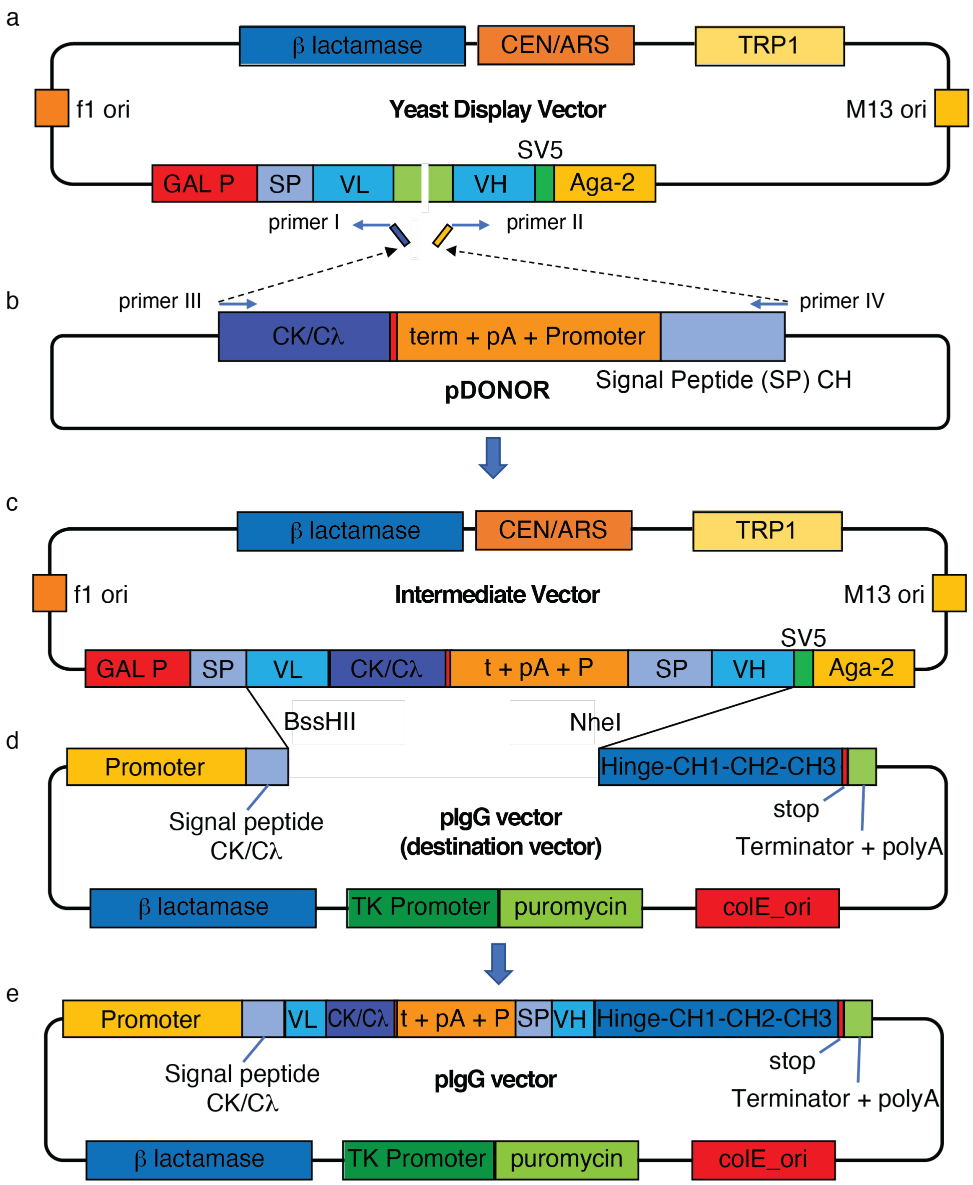

a) Inverse-PCR based amplification of the yeast display vector with a 5' primer annealing to the FW1 of the VH and 3' primer annealing to the FW4 of the VL, resulting in the linearization of the entire plasmid without the linker between VL and VH. **b)** The primers confer complementary overhangs compatible to the 5' and 3' ends of a donor fragments amplified from the pDONOR vector. The donor fragment contains the constant region of the light chains followed by translation stop and polyA site and the heavy chain promoter and signal peptide sequence**. c)** The intermediate vector is obtained by fusing the donor fragment in frame with VL and VH by NEBuilder® Assembly Kit. **d)** The cassette from the intermediate vector pool is cut out of the vector by restriction enzymes and cloned into the pIgG vector, **e**) resulting in a pool of plasmid coding for the entire LC and HC able to express IgG in mammalian cells.

Supplementary Figure 3. Description of the properties of the selected antibodies.

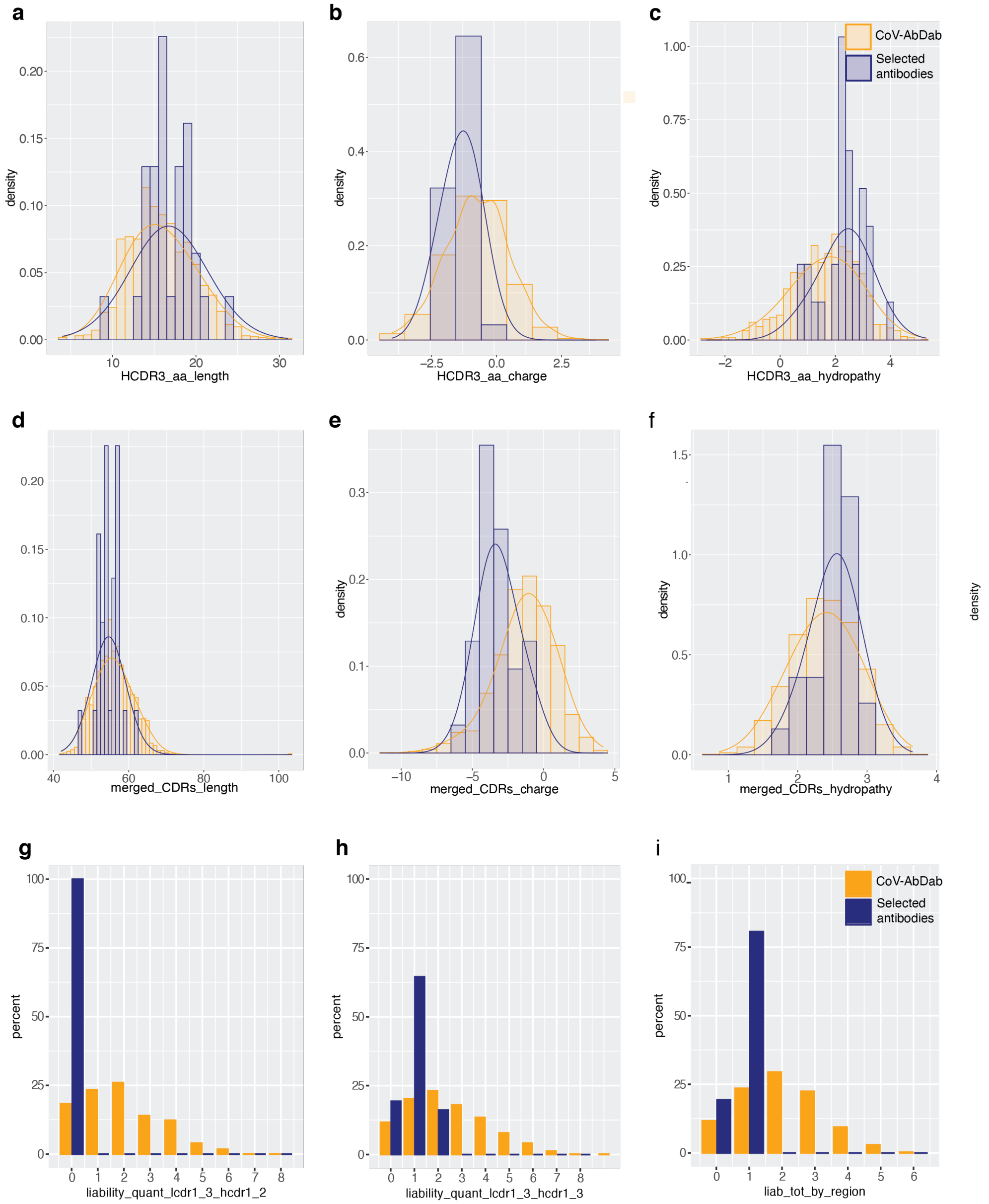

Sequence-based biophysical properties of the HCDR3 of the selected antibodies compared to antibodies in the Cov-AbDab database. **a**) HCDR3s length distribution. **b**) number of charged amino acid in the HCDR3s. **c**) hydropathy profile of the HCDR3s. **d**) Merged CDR length distribution. **e**) number of charged amino acid in the merged CDRs. **f**) Parker hydropathy profile of the merged CDRs. G - I) Sequence-based liabilities quantification across **g**) designed CDRs (H1-2, L1-3), **h**) all CDRs (H1-3, L1-3), and **i**) number of CDRs across individual (non-redundant) sequences that have ≥1 liability.

Supplementary Figure 4. Surface plasmon resonance analysis of antibody binding to SARS-CoV-2 RBD and spike protein (trimer).

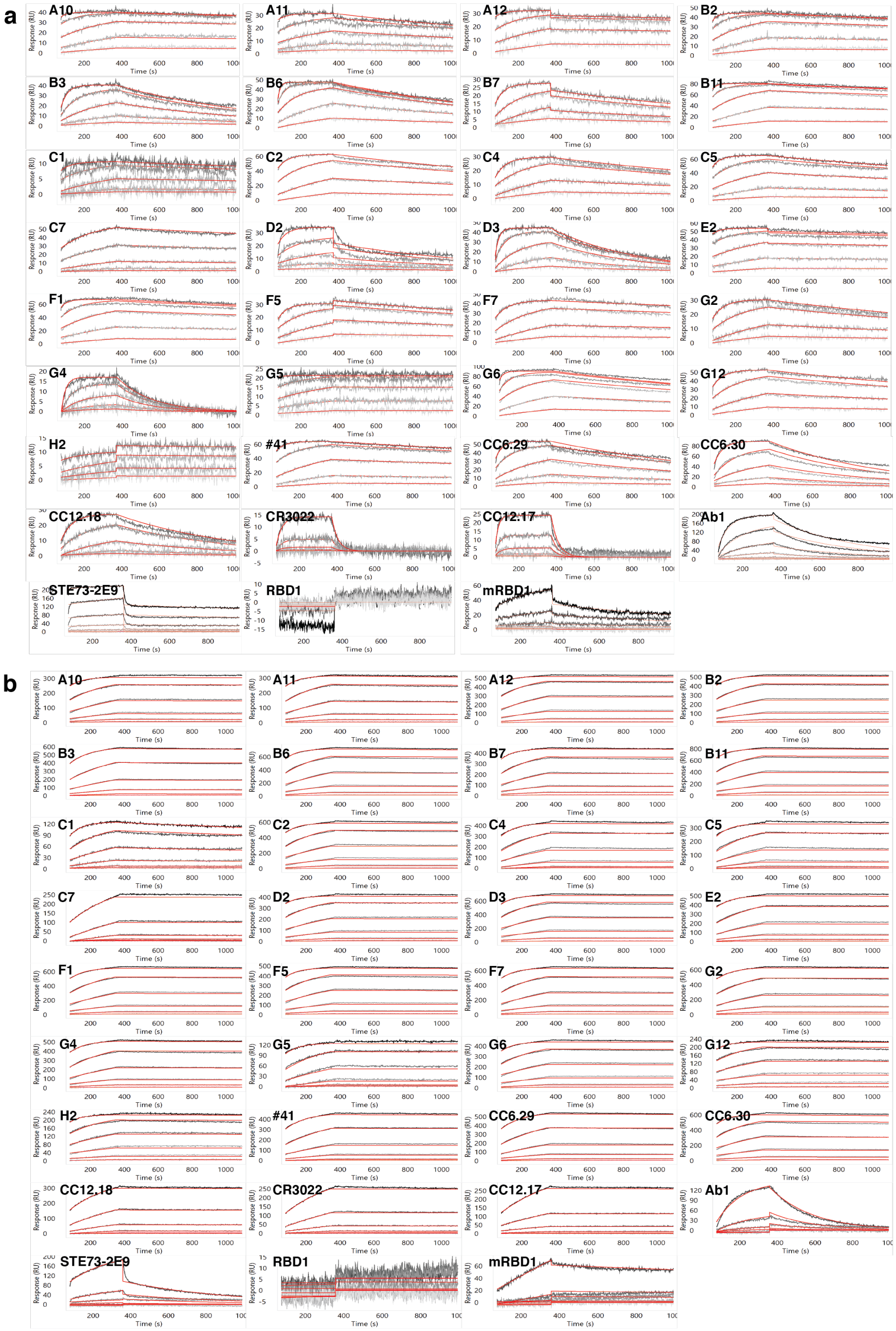

Sensorgrams of selected antibodies (including controls CC6.29, CC6.30, CC12.17, CC12.18, CR3022, Ab1, STE73-2E9, RBD1 and mRBD1.5 mAbs) showing binding to RBD **A)** and trimeric spike **B)** at different antigen concentrations.

Supplementary Figure 5. Binning SPR profiles

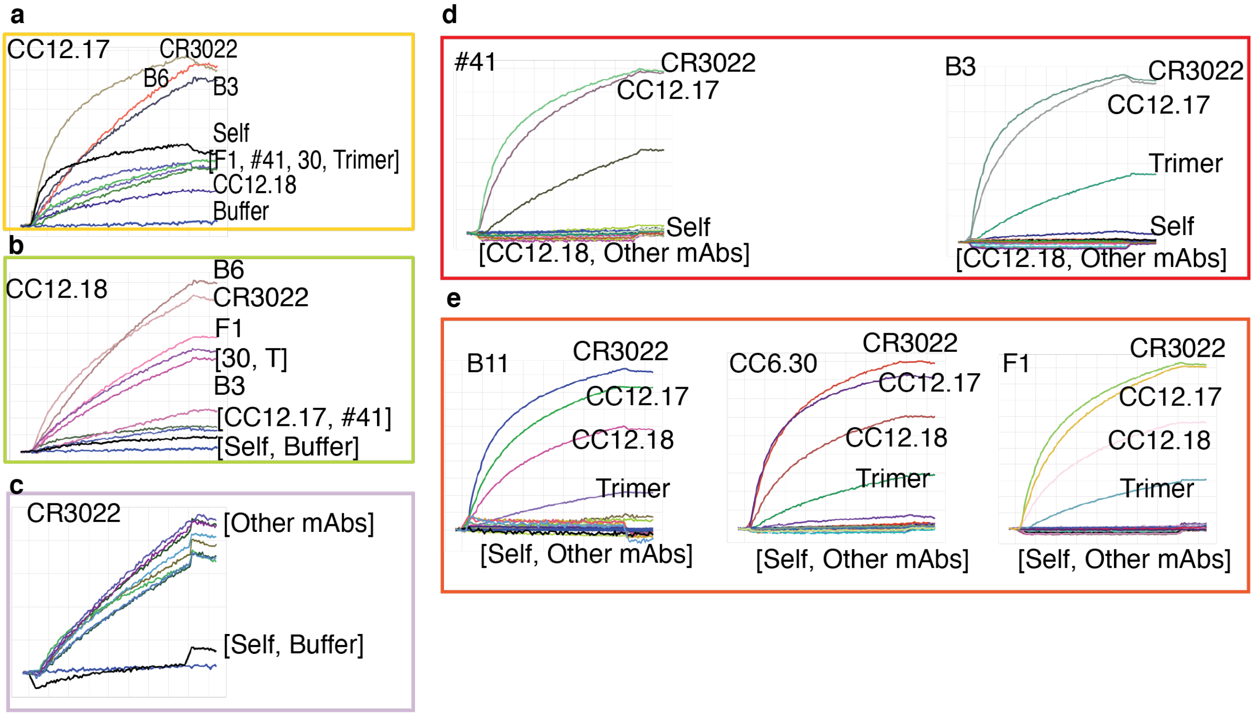

Binning SPR profiles of the CC12 antibody set: **a)** CC12.17 and **b)** CC12.18. **c)** binning group of the cross reactive CR3022 antibody **d)** different binning group for antibodies #41 and B4 **e)** Binning SPR profile of main bin group.

Supplementary Figure 6. Analysis of antibodies binding specificity.

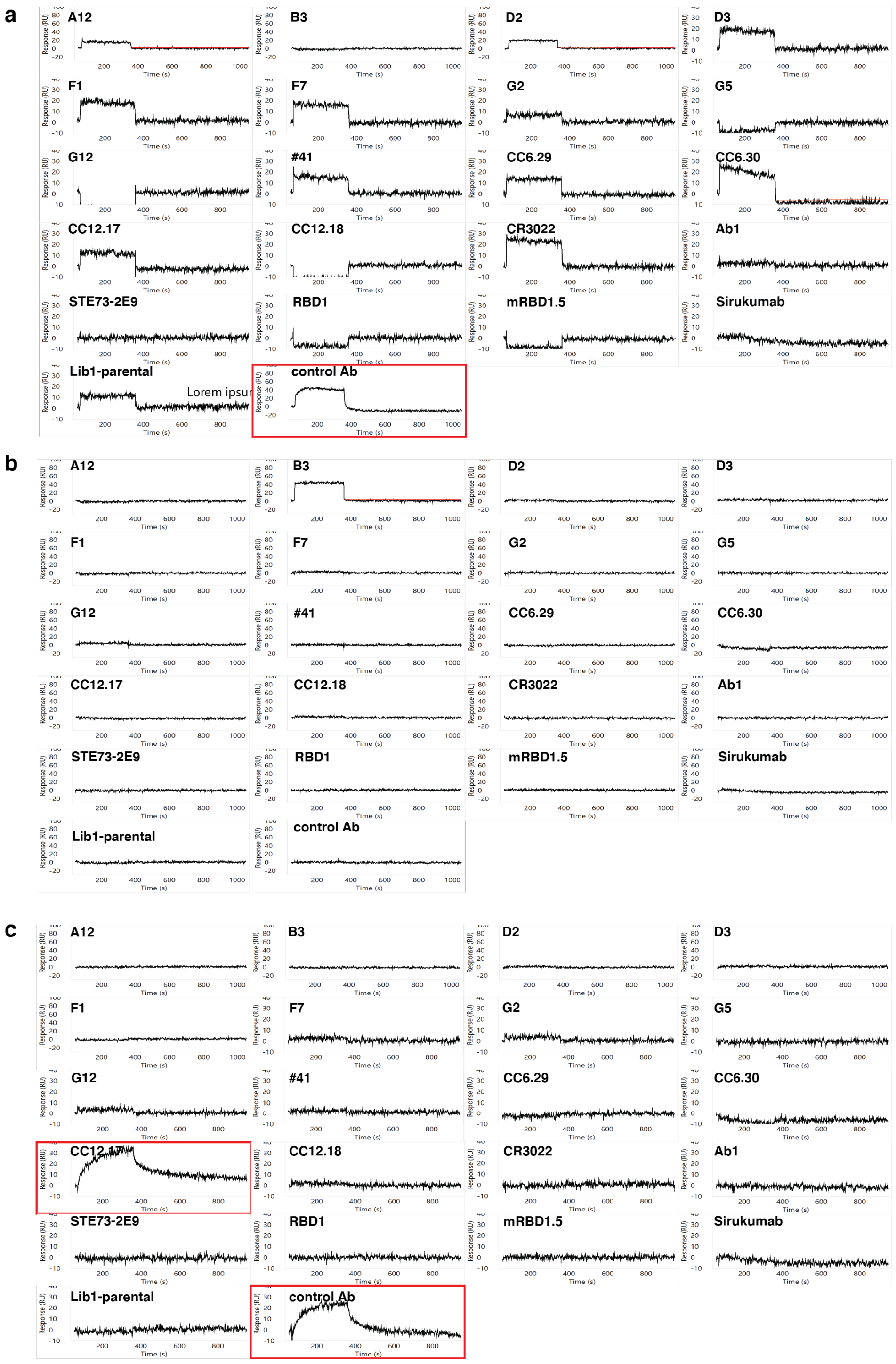

To assess the poly-reactivity of these antibodies, binding against **a)** cardiolipin, **b)** LPS and **c)** ssDNA was tested using the SPR array showing no polyspecific binding for any of the antibodies, except for CC12.17 against ssDNA, when compared to a control Ab with known polyreactive binding activity (control Ab). Antibodies showing polyspecificity are boxed red.

Supplementary Figure 7. Developability profile of selected clones.

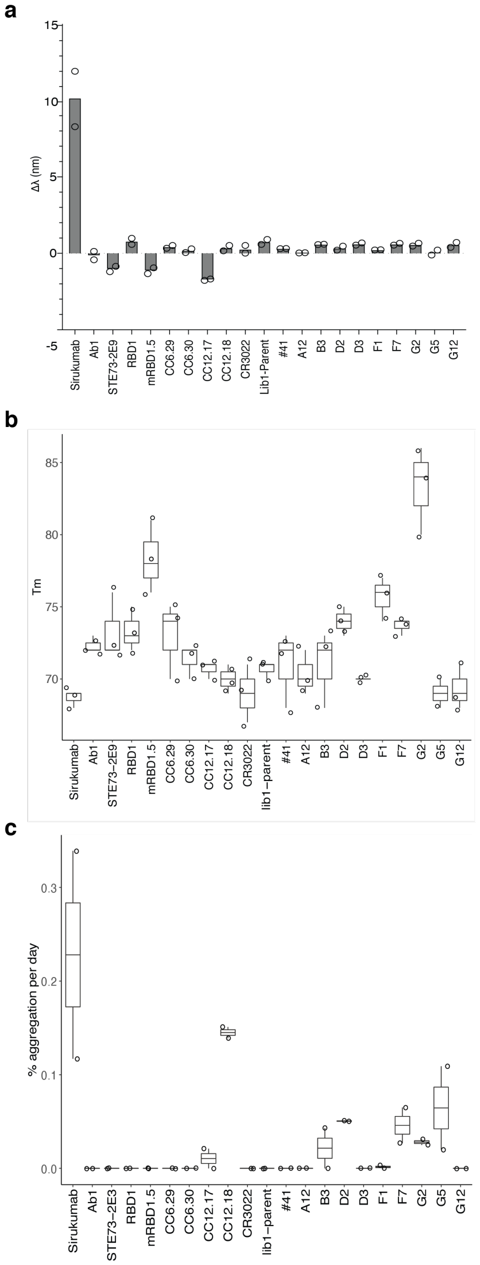

Measurements of the selected clones compared to the parental clinical scaffold (Lib1-parent), a poorly developable antibody (Sirukumab) and the other antibodies used in the study obtained from convalescent patients or other recombinant antibody libraries. **a**) AC-SINS results of wavelength shift away from PBS, data represent average of duplicates for each antibody (n=21). **b**) Tm using DSF results, data represent triplicates, boxplot metric of median, interquartile range, and max and min values (n=21). **c**) SEC assay after prolonged 37ºC exposure for 15-days, data represent %aggregation / day of duplicates, boxplot metric of median, interquartile range, and max and min values (n=21). Source data are provided as a Source Data File.

Supplementary Table 2. List of the sequence liabilities analyzed.

Aminoacid motif	modification
N[ACDEFGHIJKLMNQRSTVY][ST]	Glycosylation
N[GSTN]	Deamidation
GN[FYTG]	Deamidation
D[GSD]	Isomerization
W[GALMFWKQESVICYHRNDTP]W	Polyspecificity-WXW
RR	Polyspecificity-RR
VG	Polyspecificity-VG
VV	Polyspecificity-VV
WW	Polyspecificity-WW
Cysteine	Unpaired-Cysteine
FHW	Viscosity-Aggregation-FHW
HYF	Viscosity-Aggregation-HYF
HWH	Viscosity-Aggregation-HWH
Charge	>1

Supplementary Table 1. Sequences of the selected antibodies.

clone	VL amino acid sequence	VH amino acid sequence
**#41**	DIQMTQSPSSVSASVGDRVTITCRASRDISSYLAWY QQKPGKAPKLLIYGASTRASGVPSRFSGSGSGTDFT LTISSLQPEDFANYYCLQYSIYPWTFGGGTKVEIK	QVQLVQSGAEVKKPGASVKVSCKVSGYTSPATIHW VRQAPGKGLEWMGGISPYKGDTIYAQKFQGRVTM TEDTSTDTAYMELSSLKSEDTAVYYCAKDSYYYDS SGSGLGGGFDYWGQGTLVTVSS
**A10**	DIQMTQSPSSVSASVGDRVTITCRASRDINKYLAWY QQKPGKAPKLLIYGESSRAAGVPSRFSGSGSGTDFT LTISSLQPEDFANYYCHQYATYPWTFGGGTKVEIK	QVQLVQSGAEVKKPGASVKVSCKVSGYSFTDHSIH WVRQAPGKGLEWMGGINPNIGDTIYAQKFQGRVT MTEDTSTDTAYMELSSLKSEDTAVYYCARDGYGD YRGLYGMDVWGQGTTVTVSS
**A11**	DIQMTQSPSSVSASVGDRVTITCRASQDIGNHLAWY QQKPGKAPKLLIYASSNLASGVPSRFSGSGSGTDFT LTISSLQPEDFANYYCHQYATYPWTFGGGTKVEIK	QVQLVQSGAEVKKPGASVKVSCKVSGYSFTDHSIH WVRQAPGKGLEWMGGINPYTGDTIYAQKFQGRVT MTEDTSTDTAYMELSSLKSEDTAVYYCARDGYSYG YLSGMDVWGQGTTVTVSS
**A12**	DIQMTQSPSSVSASVGDRVTITCRASQDIGNHLAWY QQKPGKAPKLLIYASSNLASGVPSRFSGSGSGTDFT LTISSLQPEDFANYYCHQYATYPWTFGGGTKVEIK	EVQLVQSGAEVKKPGASVKVSCKVSGYSFTDHSIH WVRQAPGKGLEWMGGINPYTGDTIYAQKFQGRVT MTEDTSTDTAYMELSSLKSEDTAVYYCARDGWGS LLNGIAVAGLDYWGQGTLVTVSS
**B2**	DIQMTQSPSSVSASVGDRVTITCRASEGISNYLAWY QQKPGKAPKLLIYGTSDRASGIPSRFSGSGSGTDFTL TISSLQPEDFANYYCQQYSAFPWTFGGGTKVEIK	QVQLVQSGAEVKKPGASVKVSCKVSGYSFTDHSIH WVRQAPGKGLEWMGGINPYTGDTIYAQKFQGRVT MTEDTSTDTAYMELSSLKSEDTAVYYCARDAYSGS YPYYYYGMDVWGQGTTVTVSS
**B3**	DIQMTQSPSSVSASVGDRVTITCRASQDIGNHLAWY QQKPGKAPKLLIYASSNLASGVPSRFSGSGSGTDFT LTISSLQPEDFANYYCHQYATYPWTFGGGTKVEIK	EVQLVQSGAEVKKPGASVKVSCKVSGYTSPATIHW VRQAPGKGLEWMGGISPYKGDTIYAQKFQGRVTM TEDTSTDTAYMELSSLKSEDTAVYYCAKDSYYYDS SGSGLGGGFDYWGQGTLVTVSS
**B7**	DIQMTQSPSSVSASVGDRVTITCRASQDIGNHLAWY QQKPGKAPKLLIYASSNLASGVPSRFSGSGSGTDFT LTISSLQPEDFANYYCHQYATYPWTFGGGTKVEIK	QVQLVQSGAEVKKPGASVKVSCKVSGYSFTDHSIH WVRQAPGKGLEWMGGINPNIGDTIYAQKFQGRVT MTEDTSTDTAYMELSSLKSEDTAVYYCAKDGWGS LLNGIAVAGLDYWGQGTLVTVSS
**B11**	DIQMTQSPSSVSASVGDRVTITCRASQDIGNHLAWY QQKPGKAPKLLIYASSNLASGVPSRFSGSGSGTDFT LTISSLQPEDFANYYCHQYATYPWTFGGGTKVEIK	EVQLVQSGAEVKKPGASVKVSCKVSGYSFTDHSIH WVRQAPGKGLEWMGGINPYTGDTIYAQKFQGRVT MTEDTSTDTAYMELSSLKSEDTAVYYCARDAYPYY YGMDVWGQGTTVTVSS
**C1**	DIQMTQSPSSVSASVGDRVTITCRASQSIGSFLAWY QQKPGKAPKLLIYGESNRATGVPSRFSGSGSGTDFT LTISSLQPEDFANYYCQQYNRYPWAFGGGTKVEIK	EVQLVQSGAEVKKPGASVKVSCKVSGYSFTDHSIH WVRQAPGKGLEWMGGISTQRGDTIYAQKFQGRVT MTEDTSTDTAYMELSSLKSEDTAVYYCARDGYYGS GTYYYYGMDVWGQGTTVTVSS
**C2**	DIQMTQSPSSVSASVGDRVTITCRASEDISKYLAWY QQKPGKAPKLLIYGASNLHTGVPSRFSGSGSGTDFT LTISSLQPEDFANYYCHQYATYPWTFGGGTKVEIK	QVQLVQSGAEVKKPGASVKVSCKASGYTSPATIHW VRQAPGKGLEWMGGISPYKGDTIYAQKFQGRVTM TEDTSTDTAYMELSSLKSEDTAVYYCARDAYSSSY YYGMDVWGQGTTVTVSS
**C4**	DIQMTQSPSSVSASVGDRVTITCRASQDIGNHLAWY QQKPGKAPKLLIYASSNLASGVPSRFSGSGSGTDFT LTISSLQPEDFANYYCHQYATYPWTFGGGTKVEIK	EVQLVQSGAEVKKPGASVKVSCKVSGYSFTDHSIH WVRQAPGKGLEWMGGINPYTGDTIYAQKFQGRVT MTEDTSTDTAYMELSSLKSEDTAVYYCARDGYSGY DYGSVGRDYYYYGMDVWGQGTTVTVSS

**C7**	DIQMTQSPSSVSASVGDRVTITCRASHGISSYLAWYQQKPGKAPKLLIYDATIRATGVPSRFSGSGSGTDFTLTISSLQPEDFANYYCHQYATYPWTFGGGTKVEIK	QVQLVQSGAEVKKPGASVKVSCKVSGYSFTNYAIHWVRQAPGKGLEWMGGITPYNADTIYAQKFQGRVTMTEDTSTDTAYMELSSLKSEDTAVYYCARDGGNWAAPYSMDVWGNGTLVTVSS
**D2**	DIQMTQSPSSVSASVGDRVTITCRASRSIGNHLAWYQQKPGKAPKLLIYASSNLASGVPSRFSGSGSGTDFTLTISSLQPEDFANYYCHQYATYPWTFGGGTKVEIK	QVQLVQSGAEVKKPGASVKVSCKVSGYSFTDHSIHWVRQAPGKGLEWMGGINPYTGDTIYAQKFQGRVTMTEDTSTDTAYMELSSLKSEDTAVYYCARDARLLDYWGQGAQVTVSS
**D3**	DIQMTQSPSSVSASVGDRVTITCRASQDIGNHLAWYQQKPGKAPKLLIYASSNLASGVPSRFSGSGSGTDFTLTISSLQPEDFANYYCQQYSEYPWTFGGGTKVEIK	EVQLVQSGAEVKKPGASVKVSCKVSGYSFADYAIHWVRQAPGKGLEWMGGINPNIGDTIYAQKFQGRVTMTEDTSTDTAYMELSSLKSEDTAVYYCARDGYDFWSGSYGMDVWGQGTTVTVSS
**F1**	DIQMTQSPSSVSASVGDRVTITCRASQDIGNHLAWYQQKPGKAPKLLIYASSNLASGVPSRFSGSGSGTDFTLTISSLQPEDFANYYCHQYATYPWTFGGGTKVEIK	EVQLVQSGAEVKKPGASVKVSCKVSGYPFTSYAIHWVRQAPGKGLEWMGGITPYNADTIYAQKFQGRVTMTEDTSTDTAYMELSSLKSEDTAVYYCARDGYSSGYYGMDVWGQGTTVTVSS
**F5**	DIQMTQSPSSVSASVGDRVTITCRASQNIHSYLAWYQQKPGKAPKLLIYDTSKLQSGVPSRFSGSGSGTDFTLTISSLQPEDFANYYCHQYAAYPWTFGGGTKVEIK	EVQLVQSGAEVKKPGASVKVSCKVSGYTFSNHAIHWVRQAPGKGLEWMGGINPNIGDTIYAQKFQGRVTMTEDTSTDTAYMELSSLKSEDTAVYYCARDGYSGYVEGMDVWGQGTTVTVSS
**F7**	DIQMTQSPSSVSASVGDRVTITCRASQDIGNHLAWYQQKPGKAPKLLIYASSNLASGVPSRFSGSGSGTDFTLTISSLQPEDFANYYCHQYATYPWTFGGGTKVEIK	QVQLVQSGAEVKKPGASVKVSCKVSGYSFSTYTIHWVRQAPGKGLEWMGGINPYTGDTIYAQKFQGRVTMTEDTSTDTAYMELSSLKSEDTAVYYCARDGYSGYESSYYYYGMDVWGQGTTVTVSS
**G2**	DIQMTQSPSSVSASVGDRVTITCRASQDIGNHLAWYQQKPGKAPKLLIYGASIRESGVPSRFSGSGSGTDFTLTISSLQPEDFANYYCQQYSAFPWTFGGGTKVEIK	QVQLVQSGAEVKKPGASVKVSCKVSGYTFTANAIHWVRQAPGKGLEWMGGIGPGQGDTIYAQKFQGRVTMTEDTSTDTAYMELSSLKSEDTAVYYCARDGHCSGGSCPSGMDVWGQGTTVTVSS
**G4**	DIQMTQSPSSVSASVGDRVTITCRASQDIGNHLAWYQQKPGKAPKLLIYASSNLASGVPSRFSGSGSGTDFTLTISSLQPEDFANYYCHQYATYPWTFGGGTKVEIK	QVQLVQSGAEVKKPGASVKVSCKVSGYTLSDHAIHWVRQAPGKGLEWMGGINPYTGDTIYAQKFQGRVTMTEDTSTDTAYMELSSLKSEDTAVYYCARDGYDFWSGSYGMDVWGQGTTVTVSS
**G5**	DIQMTQSPSSVSASVGDRVTITCRASQDISHHLAWYQQKPGKAPKLLIYDASLRATGIPSRFSGSGSGTDFTLTISSLQPEDFANYYCHQYAAYPWTFGGGTKVEIK	QVQLVQSGAEVKKPGASVKVSCKVSGYSFTDHSIHWVRQAPGKGLEWMGGINPYTGDTIYAQKFQGRVTMTEDTSTDTAYMELSSLKSEDTAVYYCARDGYSYGYLSGMDVWGQGTTVTVS
**G6**	DIQMTQSPSSVSASVGDRVTITCRASQPISSYLAWYQQKPGKAPKLLIYEASKLQSGVPSRFSGSGSGTDFTLTISSLQPEDFANYYCHQYATYPWTFGGGTKVEIK	EVQLVESGAEVKKPGASVKVSCKVSGYTFSNHAIHWVRQAPGKGLEWMGGINPNIGDTIYAQKFQGRVTMTEDTSTDTAYMELSSLKSEDTAVYYCARDGYPGDYSGLDVWGQGTLVTVSS
**G12**	DIQMTQSPSSVSASVGDRVTITCRASQDIGNHLAWYQQKPGKAPKLLIYASSNLASGVPSRFSGSGSGTDFTLTISSLQPEDFANYYCHQYATYPWTFGGGTKVEIK	EVQLVQSGAEVKKPGASVKVSCKVSGYTFSNHAIHWVRQAPGKGLEWMGGINPNIGDTIYAQKFQGRVTMTEDTSTDTAYMELSSLKSEDTAVYYCARDGYGDYRGLYGMDVWGQGTTVTVSS
**H2**	DIQMTQSPSSVSASVGDRVTITCRASQGISDFLAWYQQKPGKAPKLLIYGASNRATGVPSRFSGSGSGTDFTLTISSLQPEDFANYYCHQYATYPWTFGGGTKVEIK	EVQLVQSGAEVKKPGASVKVSCKVSGYPFTRSTIHWVRQAPGKGLEWMGGINAGTGDTIYAQKFQGRVT

		MTEDTSTDTAYMELSSLKSEDTAVYYCARDGYSSGYYGMDVWGQGTLVTVSS
**E5**	DIQMTQSPSSVSASVGDRVTITCRASQDISTYLAWYQQKPGKAPKLLIYASSNLASGVPSRFSGSGSGTDFTLTISSLQPEDFANYYCHQYATYPWGFGGGTKVEIK	EVQLVQSGAEVKKPGASVKVSCKVSGYSFTDHSIHWVRQAPGKGLEWMGGINPYTGDTIYAQKFQGRVTMTEDTSTDTAYMELSSLKSEDTAVYYCAKDGWGSLLNGIAVAGLDYWGQGTLVTVSS
**G8**	DIQMTQSPSSVSASVGDRVTITCRASQSIGSFLAWYQQKPGKAPKLLIYASSNLASGVPSRFSGSGSGTDFTLTISSLQPEDFANYYCHQYATYPWTFGGGTKVEIK	EVQLVESGAEVKKPGASVKVSCKVSGYSFTDHSIHWVRQAPGKGLEWMGGINPYTGDTIYAQKFQGRVTMTEDTSTDTAYMELSSLKSEDTAVYYCARDGWGSLLNGIAVAGLDYWGQGTLVTVSS
**A2**	DIQMTQSPSSVSASVGDRVTITCRASQDIGNHLAWYQQKPGKAPKLLIYASSNLASGVPSRFSGSGSGTDFTLTISSLQPEDFANYYCHQYTTYPWTFGGGTKVEIK	QVQLVESGAEVKKPGASVKVSCKVSGYSFTDHSIHWVRQAPGKGLEWMGGINPYTGDTIYAQKFQGRVTMTEDTSTDTAYMELSSLKSEDTAVYYCARDGYCSGGSCYSFSGYGMDVWGQGTTVTVS
**H7**	DIQMTQSPSSVSASVGDRVTITCRASQDIGNHLAWYQQKPGKAPKLLIYGVSERPSGVPSRFSGSGSGTDFTLTISSLQPEDFANYYCHQYATYPWTFGDGTKVEIK	EVQLVQSGAEVKKPGASVKVSCKVSGYSFTDHSIHWVRQAPGKGLEWMGGINPYTGDTIYAQKFQGRVTMTEDTSTDTAYMELSSLKSEDTAVYYCARDGYDFWSGSYGMDVWGQGAQVTVS
**G9**	DIQMTQSPSSVSASVGDRVTITCRASQDIGNHLAWYQQKPGKAPKLLIYASSNLASGVPSRFSGSGSGTDFTLTISSLQPEDFANYYCHQYATYPWTFGGGTKVEIK	QVQLVQSGAEVKKPGASVKVSCKVSGYSFTDHSIHWVRQAPGKGLEWMGGINPYTGDTIYAQKFQGRVTMTEDTSTDTAYMELSSLKSEDTAVYYCARDGYGDYRGLYGMDVWGRGTTVTVSS
**H5**	DIQMTQSPSSVSASVGDRVTITCRASQSISTYLAWYQQKPGKAPKLLIYDASNRATGVPSRFSGSGSGTDFTLTISSLQPEDFANYYCLQYHNYPWTFGGGTKVEIK	EVQLVQSGAEVKKPGASVKVSCKVSGYSFTDHSIHWVHQAPGKGLEWMGGINPYTGDTIYAQKFQGRVTMTEDTSTDTAYMELSSLKSEDTAVYYCARDGYYGSGTYYYYGMDVWGQGTTVTVSS
**E4**	DIQMTQSPSSVSASVGDRVTITCRASQDIGNHLAWYQQKPGKAPKLLIYASSNLASGVPSRFSGSGSGTDFTLTISSLQPEDFANYYCHQYATYPWTFGGGTKVEIK	QVQLVQSGAEVKKPGASVKVSCKVSGYSFTDHSIHWVRQAPGKGLEWMGGINPYTGDTIYAQKFQGRVTMTEDTSTDTAYMELSSLKSEDTAVYYCARDGYDFWSGSYGMDVWGQGTLVTVSS
**H9**	DIQMTQSPSSVSASVGDRVTITCRASQNIDTFLAWYQQKPGKAPKLLIYASSNLASGVPSRFSGSGSGTDFTLTISSLQPEDFANYYCHQYATYPWTFGDGTKVEIK	EVQLVESGAEVKKPGASVKVSCKVSGYSFTDHSIHWVRQAPGKGLEWMGGINPYTGDTIYAQKFQGRVTMTEDTSTDTAYMELSSLKSEDTAVYYCARDAYSGSYPYYYYGMDVWGQGTTVTVSS

The antibodies highlighted in red were not further validated.
