# Supplementary material for: A pandemic-enabled comparison of discovery platforms demonstrates a naïve antibody library can match the best immune-sourced antibodies
Source: Nat Commun. 2022 Jan 24;13:462. doi: 10.1038/s41467-021-27799-z (PMC8786865; doi:10.1038/s41467-021-27799-z)
Supplement: Supplementary file 3 — Editorial Assessment Report [file 41467_2021_27799_MOESM3_ESM.pdf]

Contents of this Report

- **Manuscript details**: overview of your manuscript and the editorial team.
- **Review synthesis**: summary of the reviewer reports provided by the editors.
- **Editorial recommendation**: personalised evaluation and recommendation from all 3 journals.
- **Annotated reviewer comments**: the referee reports with comments from the editors.
- **Open research evaluation**: advice for adhering to best reproducibility practices.

About the editorial process

By selecting the **Nature Portfolio Guided Open Access option**, your manuscript was assessed for suitability in three of our titles that provide venues for publication of high-quality work across the spectrum of methods research: *Nature Methods, Nature Communications*, and *Communications Biology*. For more information about Guided Open Access, please see here.

Collaborative editorial assessment

Your editorial team discussed the manuscript to determine its suitability for the Nature Portfolio Guided OA pilot. Our assessment of your manuscript takes into account several factors, including whether the work meets the **technical standard** of the Nature Portfolio and whether the findings are of **immediate significance** to the readership of at least one of the participating journals in the Nature Portfolio Guided Open Access methods cluster.

Peer review

Experts were asked to evaluate the following aspects of your manuscript:

- **Novelty** in comparison to prior publications;
- **Likely audience** of researchers in terms of broad fields of study and size;
- **Potential impact** of the study on the immediate or wider research field;
- **Evidence** for the claims and whether additional experiments or analyses could feasibly strengthen the evidence;
- **Methodological detail** and whether the manuscript is reproducible as written;
- Appropriateness of the literature review.

Editorial evaluation of reviews

Your editorial team discussed the potential suitability of your manuscript for each of the participating journals. They then discussed the revisions necessary in order for the work to be published, keeping each journal's specific editorial criteria in mind.

Journals in the Nature portfolio will support authors wishing to transfer their reviews and (where reviewers agree) the reviewers' identities to journals outside of Springer Nature.

If you have any questions about review portability, please contact our editorial office: guidedoa@nature.com

Manuscript details

Tracking number	Submission date	Decision date
GUIDEDOA-21-00089	14 April 2021	15 June 2021

**Title**	The unique antibody discovery competition triggered by COVID-19: a naïve library directly delivering antibodies as potent as immune sources	**Corresponding author**	Andrew Bradbury<br>**Affiliation:** Specifica, Inc.
**Preprint information**	There is no preprint available for this manuscript.	**Peer review type**	Single-blind

Editorial assessment team

**Primary editor**	**Madhura Mukhopadhyay**<br>**Home Journal:** *Nature Methods*, ORCID: 0000-0003-3907-3955<br>**Email:** madhura.mukho@us.nature.com
**Editorial team members**	**Philip Loessl**, *Nature Communications*, ORCID: 0000-0001-5062-7723<br>**Anam Akhtar**, *Communications Biology*, ORCID: 0000-0002-8820-8468
**About your primary editor**	Madhura Mukhopadhyay studied microbiology as an undergraduate at Mount Carmel College in Bangalore, India, and human biology in a Master's program at the University of Copenhagen, Denmark. She obtained her Ph.D. from the Pasteur Institute in Paris, where she analyzed CD4+ T cell responses generated by a candidate HIV vaccine with Lisa Chakrabarti. Following her Ph.D., Madhura conducted postdoctoral research at Massachusetts General Hospital and University of Pennsylvania with a strong focus on deconvoluting peripheral CD4+ T cell and follicular helper T cell responses against HPV-related cancers and HIV. Madhura joined Nature Methods in March 2020.

Editorial assessment and review synthesis

**Editor's summary and assessment**	The authors have described antibodies against SARS-CoV-2 spike protein generated via a novel naïve semisynthetic library platform (method unpublished). The method involves embedding CDRs (complement determining regions that mediate antigen recognition/specificity) of the Abs from natural BCRs into clinical Ab scaffolds. The HCDR3 (heavy chain) are amplified from B cell mRNA and the remaining CDRs (LCDR1-3 and HCDR1-2) are identified by NGS of the naive repertoire, filtered using a yeast display and are synthesised on arrays. This HCDR3 is then connected to the rest using a linker and assembled into a scFv (single chain variable fragment).<br><br>In the current study, they have compared a panel of anti-S1 Abs generated via this method with Abs generated via other more traditional methods and show that the selected Abs via the semi-synthetic library have a higher neutralization potential than the controls.<br><br>Then, they compared the affinities and IC50s for the ten most potent antibodies selected here, with the single most potent antibody from every published report in the same categories (library selected/immune sourced (animals or patient derived). In each case (SPR/psuedovirus neutralization/live virus neutralization), the selected antibodies were comparable with a trend towards enhanced potential.<br><br>The strength of the paper is that it shows the potential of antibodies generated via this semi-synthetic library outperforms antibodies generated by other methods.<br><br>A weakness of this paper is that their analysis only compares to 5 other control Abs and this is not consistent across experiments. Moreover, the rationale behind choosing these controls is not clear. Finally, the method could be better validated by testing further targets.
**Editorial synthesis of reviews**	While both reviewers are in general positive about the impact and significance of the findings they raise several serious technical concerns regarding the choice of controls and lack of evidence supporting the claim of improved developability. In addition, there are also concerns about major inconsistencies in the data between the text and the figures.<br><br>For further consideration of this study at Nature Communications, the reviewer concerns mentioned above will have to be addressed in full and we would expect the authors to comply with the reviewers requests to perform aggregation/stability studies and add controls for experiments with published antibodies from naïve libraries.<br><br>For it to be considered at Communications Biology, we ask that you address all the points too, except for performing the aggregation/ stability studies to strengthen the claims on developability properties or adding additional antibodies (previously published) for your assay.

Editorial recommendation

nature methods — **Revision not invited**

While we find your study timely and of interest to our readership, we find this manuscript to be an application rather than an introduction of a new method, which precludes publication in Article (or Brief Communication) format. In addition, we find that the performance comparison of antibodies against a single target is not sufficient to justify publication *in Nature Methods* as an Analysis.

nature communications — **Major Revisions**

We feel that the study is very timely and the findings are potentially of broad interest, provided that the conclusions can be strengthened as suggested by the reviewers. We agree with the reviewers that the benefits of the newly developed antibodies need to be further demonstrated by including additional published antibodies as controls and providing stability/aggregation data that support the developability properties of the new Abs. In addition, it will be important to address all other reviewer concerns, revise the manuscript and display items accordingly.

communications biology — **Minor Revisions**

We find your study of high interest and useful to the community. While we do ask that you address all the comments of the reviewers, specifically to improve the reproducibility, add controls where prompted, explain the rationale of choosing the control antibodies and other minor comments, we are fine to waive certain referee requests. These include the requests to perform the aggregation/ stability studies (we are happy if you simply tone down the claim) and we also don't ask you to include additional antibodies isolated by other groups working with synthetic naive libraries for your assay.

Next Steps

Recommendation Summary

- **Option 1:** Revise for consideration at *Nature Communications*
- **Option 2:** Revise for consideration at *Communications Biology*

See the previous page for details

Revision

If you would like to follow our recommendation, when you are ready you can upload the revised manuscript, along with your point-by-point response to the reviewer's reports and editorial advice **at this link*.**

Revision checklist

- Cover Letter, stating to which journal you are submitting
- Revised Manuscript
- Point-by-point response to reviews
- Updated Reporting Summary and Editorial Policy Checklist
- Supplementary materials (if applicable)

Submission elsewhere

To a journal outside of Nature Portfolio

We can share the reviews with another journal outside of the Nature Portfolio if requested. You will need to request that the receiving journal office contacts us at guidedOA@nature.com. We have included editorial guidance below in the reviewer reports and open research evaluation to aid in revising the manuscript for publication elsewhere.

Annotated Reviewer Reports

The editors have included some additional comments on specific points raised by the reviewers below, to clarify requirements for publication in the recommended journal(s). However, please note that all points should be addressed in a revision, even if an editor has not specifically commented on them.

Reviewer #1

**Reviewer #1**	This reviewer has not chosen to waive anonymity. The reviewer's identity can only be shared with representatives of an established journal editorial office.
**Reviewer #1 expertise**<br>Summarised by the editor	Antibody repertoire profiling, systems immunology, protein engineering, vaccine designs, antibody development
**Editor's comments about this review**	The reviewer agrees on the impact and advance presented in this study which describes potent antibodies generated via a semi-synthetic library. They suggest that the paper would be further strengthened by the addition of more details on the construction of the phage library, as well as more data on factors such as antibody stability and aggregation.<br>A major concern highlighted by this reviewer are the inconsistencies between the data in the figures and the texts and the lack of details in the tables.

Reviewer #1 comments

**Overview**	**Overall significance -** The SARS-CoV-2 global pandemic has resulted in many research groups attempting to isolate antibodies against SARS-CoV-2 using a wide variety of different methods. In this manuscript, the authors describe the use of their semi-synthetic naïve scFv library to screen for scFvs that bind to the S1 domain of SARS-CoV-2 spike protein. The library used in the study is constructed using their patented platform that combines antibody scaffolds from clinical mAbs with CDRs (except CDRH3) from BCRs sampled from multiple donors that have sequence liabilities removed to improve the developability properties. The CDRH3 diversity is generated via amplification directly from BCR mRNA. Starting with this semi-synthetic library, the authors completed 2 rounds of phage panning before transferring the screened scFv variants into a yeast display system to do 3 additional rounds of FACS sorting. Subsequently, they sequenced 96 clones, which resulted in 31 unique sequences, and they selected 23 to characterize their functional features as full-length IgGs.

	Their principal finding is that some of the antibodies they isolated from their naïve scFv library demonstrate extremely high binding affinities (as low as 34 pM to RBD and 38 pM to S trimer) with potent neutralization activities in their lentivirus pseudovirus assay (IC50 of 1.7 ng/mL) as well as live virus assay using Vero cells (IC50 of 1.3 ng/mL). The authors used previously published 5 mAb isolated from B cell profiling of Covid-19 patients as a benchmark, and several of their antibodies demonstrated greater potency than those reference mAbs.<br><br>These antibodies represent the best antibodies that have been isolated from naïve libraries, in terms of binding affinities and neuralization activities. Their findings are of great significance that demonstrates the impressive potential of the semi-synthetic library they have constructed, especially given their focus on the developability features of the library.

Specific comments

#	Reviewer comment	Editorial comment
1	**Impact -** One potential drawback of a synthetic library-based antibody discovery approach is the lower developability of selected antibodies. The authors address this through their generation 3 antibody library designed to eliminate CDRs with sequence liabilities, and they also demonstrate the antibodies selected from the library having comparable potency as antibodies isolated from B cells of convalescent patients.	
2	**Strength of the claims -** The technical approach for mAb characterization, including detailed binding and neutralization assays, is robust and convincing. On the other hand, there are some key concerns/questions, which are further discussed below (as well as in the "Reproducibility" section).	
3	**Construction of the naïve library:**<br><br>It would strengthen the manuscript to include additional details regarding the construction of the initial phage library used for screening (some of the information included in the accompanying manuscript).	For consideration at *Nature Communications*, it will be important to provide these details.<br><br>The details for construction of phage library as requested here is important for *Communications Biology* as well.

**4**	**Developability properties:**<br>While the authors have checked for poly-specificity and sequence liabilities of the selected antibodies, but additional measurements into stability/aggregation (e.g., Tm measurement, SEC, etc.) would further strengthen their claim that naïve antibody libraries can effectively compete with B cell profiling to provide therapeutic antibodies without further downstream optimization.	*Nature Communications* would expect you to perform the requested stability/aggregation measurements and address the minor points.<br>For *Communications Biology,* kindly clarify only the minor points asked here. We do not require the authors to perform the additional measurements into stability, aggregation etc. as requested.
5 – 6: minor points related to comment #4		
**5**	Sequencing the 96 clones selected at the end of the screening process resulted in 31 unique sequences. Did the analysis included in Supplementary Figure 2 consider all 31 sequences or the 23 sequences that were selected for further characterization? Figure 2 should include all 31 sequences.	
**6**	Can the authors describe how the 23 antibodies were selected for subsequent biochemical and functional assays? Were all 31 antibodies cloned and tested as IgG but 8 were removed because they didn't convert to the IgG format? The authors should discuss.	
**7**	**Control antibodies:**<br>CC6.29, CC6.30 (targeting RBD-A), CC12.17, CC12.18 (targeting RBD-B), and CR3022 (targeting RBD-C) were selected as 5 control antibodies to benchmark the antibodies selected from their library. Based on the data presented in the Rogers et al study where these antibodies were characterized, it appears that they were chosen for having high neutralization activities (except CR3022), although they do not have the highest binding affinities. Authors should discuss their rationales for choosing CC6.29/30 for RBD-A and CC12.17/18 for RBD-B. Also, the authors should consider including a couple of previously published antibodies isolated from naïve libraries in their assays.	For *Nature Communications*, please include published antibodies from naive libraries as additional controls and explain how the control antibodies were selected.<br>For *Communications Biology*, kindly explain the rationale behind choosing the five antibodies. We do not ask you to repeat your assay with more previously published antibodies, as requested here.

**8**	**Figure 5** should not include any binding data from antibodies evaluated in non-IgG format (i.e. Fab and scFv) for consistent and unbiased comparisons.	This point must be addressed for both *Nature Communications* and *Communications Biology.*
**9**	**Reproducibility -** Data description (a major concern):<br><br>There are numerous inconsistencies regarding how the binding and neutralization data are described in the main texts and presented in Figure 2A/Table 1, which are detailed below. For clarity, the authors should correct any inconsistencies.<br><br>For clarity, the authors should correct any inconsistencies.<br><br>• Line 116-7: The avidities of the selected antibodies to trimeric spike range from 38 pM to 32.7 nM (not 37 pM to 32 nM) according to Figure 2A. The median values also appear to be inconsistent with the data in Figure 2A.<br>• Line 117-8: 399 pM and 242 pM need to be switched.<br>• Line 118-9: According to Figure 2A, the binding affinities of CC12.17, CC12.18, and CR3022 to RBD are 84.6 nM, 2.2 nM, and 11 nM, respectively, but the texts says “1.6 nM, ≥50 nM and 11 nM”.<br>• Line 142: According to Table 1, IC50 values ranges from 1.7 to 85.2 ng/mL, not 1.7 to 92 ng/mL.	For further consideration at either *Nature Communications* or *Communications Biology*, it will be essential to address these concerns in full.
**10**	Some of the figures are not legible (e.g., Supplementary Figures 3 and 4). Please edit those figures.	
**11**	Supplementary Figure 6 is not referenced in the manuscript.	
**12**	While Figure 4D nicely shows the ratios of neutralization activities of the selected mAbs against the UK and SA variants, please include the neutralization IC50 values for each.	
**13**	Table 2, please include antibody names next to the neutralization values included in the table.	

Reviewer #2

**Reviewer #2**	This reviewer has not chosen to waive anonymity. The reviewer's identity can only be shared with representatives of an established journal editorial office.
**Reviewer #2 expertise** Summarised by the editor	Antibody engineering, protein therapeutics, adaptive immune responses, antibody repertoire analysis
**Editor's comments about this review**	While this reviewer is also positive about the potential impact of this study, they are concerned by the tone of the paper which suggests a competition of antibody discovery methods. Like reviewer #1, they also recommend addition of more data to evaluate antibody stability and aggregation as well as further discussion of the CDR3 features.

Reviewer #2 comments

**Overview**	**Overall significance -** Ferrara et al describe the isolation of panel of high affinity and potently neutralizing SARS-CoV2antibodies from a large library of naive scFvs using a combination of phage and yeast display. The authors report the successful isolation of a relatively large panel of high affinity (KD<100 pM) antibodies some of which were shown to be neutralizing and importantly a few also neutralized VOCs including B.1.351. These are interesting and timely findings and point to the utility of the author's naive library and screening and IgG characterization processes.

Specific comments

#	Reviewer comment	Editorial comment
1	Having said that what I find objectionable in the paper is the casting of the story as a "competition" for SARS-CoV2 antibodies compared to other antibody discovery efforts. Frankly, this is pointless and a thinly veiled corporate propaganda. It makes little sense to compare different methods for isolation antibodies that were performed by different labs using very different approaches and with different objectives. For many of the studies referenced here the emphasis was not isolating the highest affinity clones to begin with.	

**2**	Furthermore, why is the isolation of high affinity antibodies from a naive library deemed to be more significant (ie "winning" the "competition") than using screening followed by affinity maturation. For example, using naive libraries + affinity maturation Kossiakoff and coworkers (ref 41) and others have found low pM affinity, neutralizing antibodies.<br><br>Before publication it is critical that the paper is revised to recast the storyline and eliminate any mention of competition and simply present the findings for what they are.<br><br>Additionally the authors should address the following technical points:	While we realize that this comment is rather harsh in tone, we that the claims regarding an "antibody competition" may distract readers from the value of this work. For *Nature Communications*, we would therefore ask you to substantially tone down or remove these claims.
**Major**		
**3**	Antibody sequences must be shown. Also some analysis and brief discussion of the CDR3s (length, aa composition etc) should be presented.	Both of these points will have to be addressed for *Nature Communications.* It will be important to provide additional aggregation/stability data that support the developability claims.<br><br>For *Communications Biology*, please address the first point, regarding adding further analysis on CDR3s. As mentioned previously with R3 (Reviewer #1's comment), we are fine if you tone down the developability claims, without requiring antibody aggregation and stability evaluation.
**4**	The authors argue that the antibodies isolated are not likely to have developability liabilities. However, this is based solely on sequence analysis and sidesteps perhaps the most critical issues, propensity to aggregation and stability. The authors should at the very least present SEC data to evaluate propensity for aggregation for at least a few of the IgGs (say n=5-6) as well as some stability data.	
**Minor**		
**5**	Table 2: The Table does not have the antibodies for which the EC50 values are reported!	Please edit Table 2 accordingly and add the requested control experiments for *Nature Communications.*<br><br>For *Communications Biology,* it is important to please edit the table and the control asked here.
**6**	SI Fig 4: The polyreactivity SPR data have no positive control! Because avidity is important in polyreactivity assays ELISA may be more relevant (but not required for the paper).	
**7**	**Reproducibility -** Need to provide the sequences of the antibodies for reproducibility.	

Open Research Evaluation

Data availability

Data Availability statement

Thank you for including a Data Availability statement. However, we noted that you have only indicated that data are available upon request. The data availability statement must make the conditions of access to the “minimum dataset” that are necessary to interpret, verify and extend the research in the article, transparent to readers.

In addition, Nature Portfolio policies include a strong preference for research data to be archived in public repositories. For data types without specific repositories, we recommend that data are deposited in a generalist repository such as figshare or Dryad. More information about our data availability policy can be found here: https://www.nature.com/nature-portfolio/editorial-policies/reporting-standards#availability-of-data

See here for more information about formatting your Data Availability Statement: http://www.springernature.com/gp/authors/research-data-policy/data-availability-statements/12330880

In addition, please make antibody sequence data available.

Please cite the link to your publicly available dataset in the reference list

Mandatory data deposition

For your DNA sequencing data, submission to a community-endorsed, public repository is mandatory for publication in a Nature Portfolio journal and is best practice for publication in any venue. Accession numbers must be provided in the paper. Examples of appropriate public repositories are listed below:

- GenBank
- Sequence Read Archive (WGS or WES data)
- The European Nucleotide Archive (ENA)

For more information on mandatory data deposition policies at the Nature Portfolio, please visit http://www.nature.com/authors/policies/availability.html#data

For an up-to-date list of approved repositories for each mandatory data type, please visit https://www.springernature.com/gp/authors/research-data-policy/repositories/12327124

Other data requests

All source data underlying the graphs and charts presented in the main figures must be made available as Supplementary Data (in Excel or text format) or via a generalist repository (eg, Figshare or Dryad). This is mandatory for publication in a Nature Portfolio journal, but is also best practice for publication in any venue.

Data publishing recommendation

You may also be interested in publishing a Data Note with BMC Research Notes to improve the usability of your data set. See the journal website for details: https://bmcresnotes.biomedcentral.com/

For more information about data publishing at Springer Nature, please see https://www.springernature.com/gp/authors/research-data/research-data-publishing

Code availability and citation

Please include a statement under the heading "Code Availability", indicating whether and how the custom code/software reported in your study can be accessed, including any restrictions to access. This section should also include information on the versions of any software used, if relevant, and any specific variables or parameters used to generate, test, or process the current dataset. Code availability statements should be provided as a separate section after the Data Availability section.

Upon publication, Nature Portfolio journals consider it best practice to release custom computer code in a way that allows readers to repeat the published results. Code should be deposited in a DOI-minting repository such as Zenodo, Gigantum or Code Ocean and cited in the reference list following the guidelines described in our policy pages (see link below). Authors are encouraged to manage subsequent code versions and to use a license approved by the open source initiative.

Full details about how the code can be accessed and any restrictions must be described in the Code Availability statement.

See here for more information about our code availability policies: https://www.nature.com/nature-portfolio/editorial-policies/reporting-standards#availability-of-computer-code

We also provide a Code and Software submission checklist that you may find useful: https://www.nature.com/documents/nr-software-policy.pdf

Please note: because of advanced features used in this form, you must use Adobe Reader to open the documents and fill it out.

Materials availability

We encourage you to include within the Data Availability statement whether these antibodies can be made available to readers. If so, please also include the email address for requests.

Reporting and reproducibility

Reproducibility

Nature Portfolio journals allow unlimited space for Methods. The Methods must contain sufficient detail such that the work could be repeated. It is preferable that all key methods be included in the main manuscript, rather than in the Supplementary Information. Please avoid use of "as described previously" or similar, and instead detail the specific methods used with appropriate attribution.

Statistics

Please see the 'extended comments' file.

Data presentation

The quality of some of the figures appears to be quite low. If possible, we suggest replacing these with higher-resolution images (eg Supp figs 3 & 4).

Other notes

Please include a gating strategy for all flow cytometry experiments.

We have included as an attachment to the decision letter a version of your Reporting Summary with a few notes. This is mainly for your information, but we hope it is helpful when preparing your revised manuscript. If you decide to resubmit the manuscript for further consideration, please be sure to include an updated Reporting Summary.
