# Supplementary material for: A pandemic-enabled comparison of discovery platforms demonstrates a naïve antibody library can match the best immune-sourced antibodies
Source: Nat Commun. 2022 Jan 24;13:462. doi: 10.1038/s41467-021-27799-z (PMC8786865; doi:10.1038/s41467-021-27799-z)
Supplement: Supplementary file 4 — Description of Additional Supplementary Files [file 41467_2021_27799_MOESM4_ESM.docx]

**File Name:** Supplementary Data 1.

**Description:** Primers used in this study.

-pDan5topDNL6_5’ and pDan5topDNL6_3’ are the primers used to subclone the phage output into the yeast display vector by homologous recombination directly into yeast cells.

- Gen3-JK-CK, Gen3-JL-CL, Abr_SP, Ani_Evo_SP, Cre_SP, Mep_SP, Nec_SP are the primers used to amplify the cassette from the pDONOR vectors.

- Gen3-JK-CK_INV, Gen3-JL-CL_INV, Abr_ invPCR, Ani_Evo_ invPCR, Cre_ invPCR, Mep_ invPCR, Nec_ invPCR are the primers used to amplify by inverse PCR the selected antibodies.
