# Supplementary material for: A pandemic-enabled comparison of discovery platforms demonstrates a naïve antibody library can match the best immune-sourced antibodies
Source: Nat Commun. 2022 Jan 24;13:462. doi: 10.1038/s41467-021-27799-z (PMC8786865; doi:10.1038/s41467-021-27799-z)
Supplement: Supplementary file 6 — Reporting Summary [file 41467_2021_27799_MOESM6_ESM.pdf]

nature portfolio

Corresponding author(s): Andrew Bradbury

Last updated by author(s): December 3 2021

Reporting Summary

Nature Portfolio wishes to improve the reproducibility of the work that we publish. This form provides structure for consistency and transparency in reporting. For further information on Nature Portfolio policies, see our Editorial Policies and the Editorial Policy Checklist.

Statistics

For all statistical analyses, confirm that the following items are present in the figure legend, table legend, main text, or Methods section.

n/a	Confirmed	
☐	☒	The exact sample size (*n*) for each experimental group/condition, given as a discrete number and unit of measurement
☐	☒	A statement on whether measurements were taken from distinct samples or whether the same sample was measured repeatedly
☒	☐	The statistical test(s) used AND whether they are one- or two-sided<br>*Only common tests should be described solely by name; describe more complex techniques in the Methods section.*
☐	☒	A description of all covariates tested
☐	☒	A description of any assumptions or corrections, such as tests of normality and adjustment for multiple comparisons
☐	☒	A full description of the statistical parameters including central tendency (e.g. means) or other basic estimates (e.g. regression coefficient) AND variation (e.g. standard deviation) or associated estimates of uncertainty (e.g. confidence intervals)
☒	☐	For null hypothesis testing, the test statistic (e.g. *F*, *t*, *r*) with confidence intervals, effect sizes, degrees of freedom and *P* value noted<br>*Give P values as exact values whenever suitable.*
☒	☐	For Bayesian analysis, information on the choice of priors and Markov chain Monte Carlo settings
☒	☐	For hierarchical and complex designs, identification of the appropriate level for tests and full reporting of outcomes
☒	☐	Estimates of effect sizes (e.g. Cohen's *d*, Pearson's *r*), indicating how they were calculated

Our web collection on statistics for biologists contains articles on many of the points above.

Software and code

Policy information about availability of computer code

Data collection	No software was used for data collection
Data analysis	Surface Plasmon Resonance data were analyzed using the Carterra LSA software Kinetics 1.7.2.3202.<br>Binning analysis was performed using he Carterra LSA software Epitope V.1.7.1.3055.<br>Graphs and statistical analyses were performed in GraphPad Prism 9.2.0 and R.<br>Figure were prepared using GraphPad 9.2.0, R and Adobe Illustrator.

For manuscripts utilizing custom algorithms or software that are central to the research but not yet described in published literature, software must be made available to editors and reviewers. We strongly encourage code deposition in a community repository (e.g. GitHub). See the Nature Portfolio guidelines for submitting code & software for further information.

Data

Policy information about availability of data

All manuscripts must include a data availability statement. This statement should provide the following information, where applicable:

- Accession codes, unique identifiers, or web links for publicly available datasets
- A description of any restrictions on data availability
- For clinical datasets or third party data, please ensure that the statement adheres to our policy

The sequences of the light and heavy chain variable regions of the selected antibodies can be found in the Supplementary Information and on Genebank (MZ927183- MZ927184- MZ927185- MZ927186- MZ927187- MZ927188- MZ927189- MZ927190- MZ927191- MZ927192- MZ927193- MZ927194- MZ927195- MZ927196- MZ927197- MZ927198- MZ927199- MZ927200- MZ927201- MZ927202- MZ927203- MZ927204- MZ927205- MZ927206- MZ927207- MZ927208- MZ927209- MZ927210- MZ927211- MZ927212- MZ927213- MZ927214- MZ927215- MZ927216- MZ927217- MZ927218- MZ927219- MZ927220- MZ927221-

MZ927222- MZ927223- MZ927224- MZ927225- MZ927226- MZ927227-MZ927228). Those sequences can also be found on Figshare (10.6084/m9.figshare.17004013
) and on Zenodo (https://doi.org/10.5281/zenodo.5686448). For Supplementary Figure 3 we used the CoV-AbDab (http://opig.stats.ox.ac.uk/webapps/covabdab/), while for Figure 5 we access data from the dataset published by CoVIC (https://covic.lji.org/).
Source data are provided with this paper.

Field-specific reporting

Please select the one below that is the best fit for your research. If you are not sure, read the appropriate sections before making your selection.

- [x] Life sciences
- [ ] Behavioural & social sciences
- [ ] Ecological, evolutionary & environmental sciences

For a reference copy of the document with all sections, see nature.com/documents/nr-reporting-summary-flat.pdf

Life sciences study design

All studies must disclose on these points even when the disclosure is negative.

Sample size	N/A the manuscript analyze antibodies obtained from a semi synthetic recombinant library
Data exclusions	No data were excluded
Replication	All experiments were successfully replicated and the numbers are indicated in the figure legends
Randomization	The study described isolation and characterization of antibodies derived from a semi synthetic recombinant library. There was no step where randomization was relevant.
Blinding	Blinding is not relevant to such kind of study, since it was not a clinical research study

Reporting for specific materials, systems and methods

We require information from authors about some types of materials, experimental systems and methods used in many studies. Here, indicate whether each material, system or method listed is relevant to your study. If you are not sure if a list item applies to your research, read the appropriate section before selecting a response.

Materials & experimental systems

n/a	Involved in the study	
☐	☒	Antibodies
☐	☒	Eukaryotic cell lines
☒	☐	Palaeontology and archaeology
☒	☐	Animals and other organisms
☒	☐	Human research participants
☒	☐	Clinical data
☒	☐	Dual use research of concern

Methods

n/a	Involved in the study	
☒	☐	ChIP-seq
☐	☒	Flow cytometry
☒	☐	MRI-based neuroimaging

Antibodies

Antibodies used	a) V5 Tag Monoclonal Antibody (2F11F7) ThermoFisher #37-7500 conjugated in house with R-Phycoerythrin PE / R-Phycoerythrin Conjugation Kit - Lightning-Link® (ab102918)<br>b) Anti-human Fc (Southern Biotech, #2048-01)<br>c) Anti-M13 Antibody, HRP conjugated, mouse monoclonal (SinoBiological, 11973-MM05T-H)
Validation	a) The V5 Tag Monoclonal Antibody conjugated with PE has been widely used by our group and other. Here are some of our citation were the antibody has been previously validate:<br>- Ferrara, F., et al., Using phage and yeast display to select hundreds of monoclonal antibodies: application to antigen 85, a tuberculosis biomarker. PLoS One, 2012. 7(11): p. e49535.<br>- Ferrara, F., et al., Recombinant renewable polyclonal antibodies. MAbs, 2015. 7(1): p.32-41.<br>An anti-human Fc (Southern Biotech, #2048-01) has been successfully used for conjugation of human antibodies on SRP chips Kaymakcalan Z, Sakorafas P, Bose S, Scesney S, Xiong L, Hanzatian DK, et al. Comparisons of affinities, avidities, and complement activation of adalimumab, infliximab, and etanercept in binding to soluble and membrane tumor necrosis factor. Clin Immunol. 2009;131:308-16. (Surface Plasmon Resonance).<br>b) The anti-human Fc has been validated by several other groups, and here are the references mentioned in the provider website.<br>- Wang Q, Chen K, Liu F, Zhao F, Gupta S, Zhang N, et al. Novel GLP-1 fusion chimera as potent long acting GLP-1 receptor agonist. PLoS One. 2010;5(9):e12734. (ELISA)

- Shen X, Hu G, Jiang S, He F, Xing W, Li L, et al. Engineering and characterization of a baculovirus-expressed mouse/human chimeric antibody against transferrin receptor. Protein Eng Des Sel. 2009;22:723-31. (WB)
- Djoumerska-Alexieva IK, Dimitrov JD, Voynova EN, Lacroix-Desmazes S, Kaveri SV, Vassilev TL. Exposure of IgG to an acidic environment results in molecular modifications and in enhanced protective activity in sepsis. FEBS J. 2010;277:3039-50. (WB)
- Kaymakcalan Z, Sakorafas P, Bose S, Scesney S, Xiong L, Hanzatian DK, et al. Comparisons of affinities, avidities, and complement activation of adalimumab, infliximab, and etanercept in binding to soluble and membrane tumor necrosis factor. Clin Immunol. 2009;131:308-16. (Surface Plasmon Resonance)

c) Anti-M13-HRP from Sinobiological is now the antibody of choice for experiments where the detection of M13 phage is necessary, such as phage-ELISA, replacing the discontinued one from GE. Here are some of the publications reported in the provider website:
- Messing, J. et al., 1993, Methods Mol. Biol. 23: 9-22.
- Mori, K. et al., 1996, Antiviral Res. 31 (1-2): 79-86.
- Sidhu, S.S. et al., 2001, Biomol Eng. 18 (2): 57-63.
- Sitohy, M. et al., 2006, J Agric Food Chem. 54 (11): 3800-6.
- Khalil, A.S. et al., 2007, Proc Natl Acad Sci. USA. 104 (12): 4892-7.

Eukaryotic cell lines

Policy information about cell lines

Cell line source(s)	Saccharomyces cerevisiae Meyen ex E.C. Hansen (ATCC MYA-4941) strain EBY100<br>HEK293T cells for pseudovirus based experiments (ATCC CRL-3216).<br>Expi293F™ Cells for antibody expression (ThermoFisher A14527).<br>Vero E6 cells for the experiment involving the live virus (ATCC CRL-1586).<br>HeLa cells for the ACE2 expression (ATCC CCL-2)
Authentication	The cell lines were authenticated by ATCC and ThermoFisher by using STR profiling.
Mycoplasma contamination	Cells were always negative for mycoplasma contamination
Commonly misidentified lines<br>(See ICLAC register)	No commonly misidentified cell lines were used in the study.

Flow Cytometry

Plots

Confirm that:

- [x] The axis labels state the marker and fluorochrome used (e.g. CD4-FITC).
- [x] The axis scales are clearly visible. Include numbers along axes only for bottom left plot of group (a 'group' is an analysis of identical markers).
- [x] All plots are contour plots with outliers or pseudocolor plots.
- [x] A numerical value for number of cells or percentage (with statistics) is provided.

Methodology

Sample preparation	Yeast cells were washed by adding washing buffer (PBS, 0.5% BSA and 50 nM EDTA), spin down and buffer was removed. Cells were incubated 30 min with the antigen solution, washed again, incubated with the anti-V5 antibody and streptavidin-Alexa-Fluor-633, washed again and analyzed.
Instrument	BD FASCAria III
Software	BD Diva, FlowJo
Cell population abundance	Each yeast sample consisted of at least 10^6 cells.
Gating strategy	Double positive yeast cells were sorted: positive for expression (PE signal on the X-axis) and display of the scFVs on their surface detectable with V5-PE antibody, and positive for the binding of the biotinylated antigen by the scFvs detected using fluorescently labeled streptavidin (APC signal on the Y-axis).

- [x] Tick this box to confirm that a figure exemplifying the gating strategy is provided in the Supplementary Information.
